# Phenotypic Switching Induced by Damaged Matrix Is Associated with DNA Methyltransferase 3A (DNMT3A) Activity and Nuclear Localization in Smooth Muscle Cells (SMC)

**DOI:** 10.1371/journal.pone.0069089

**Published:** 2013-08-07

**Authors:** Jia-Xin Jiang, Karen J. Aitken, Chris Sotiropolous, Tyler Kirwan, Trupti Panchal, Nicole Zhang, Shuye Pu, Shoshana Wodak, Cornelia Tolg, Darius J. Bägli

**Affiliations:** 1 Developmental and Stem Cell Biology, Hospital for Sick Children, Toronto, Ontario, Canada; 2 Division of Urology, Hospital for Sick Children, Toronto, Ontario, Canada; 3 Department of Physiology, University of Toronto, Toronto, Ontario, Canada; 4 Cell and Systems Biology, University of Toronto, Toronto, Ontario, Canada; 5 Centre for Computational Medicine, Hospital for Sick Children, Toronto, Ontario, Canada; 6 Institute of Medical Sciences, Department of Surgery, University of Toronto, Toronto, Ontario, Canada; University Medical Center Utrecht, Netherlands

## Abstract

Extracellular matrix changes are often crucial inciting events for fibroproliferative disease. Epigenetic changes, specifically DNA methylation, are critical factors underlying differentiated phenotypes. We examined the dependency of matrix-induced fibroproliferation and SMC phenotype on DNA methyltransferases. The cooperativity of matrix with growth factors, cell density and hypoxia was also examined. Primary rat visceral SMC of early passage (0–2) were plated on native collagen or damaged/heat-denatured collagen. Hypoxia was induced with 3% O_2_ (balanced 5% CO_2_ and 95% N_2_) over 48 hours. Inhibitors were applied 2–3 hours after cells were plated on matrix, or immediately before hypoxia. Cells were fixed and stained for DNMT3A and smooth muscle actin (SMA) or smooth muscle myosin heavy chain. Illumina 450 K array of CpG sites was performed on bisulfite-converted DNA from smooth muscle cells on damaged matrix vs native collagen. Matrix exquisitely regulates DNMT3A localization and expression, and influences differentiation in SMCs exposed to denatured matrix +/− hypoxia. Analysis of DNA methylation signatures showed that Matrix caused significant DNA methylation alterations in a discrete number of CpG sites proximal to genes related to SMC differentiation. Matrix has a profound effect on the regulation of SMC phenotype, which is associated with altered expression, localization of DNMTs and discrete changes DNA methylation.

## Introduction

Matrix composition affects cell behavior through many mechanisms, such as receptor-mediated signaling [1]–[5], cytoskeletal tension [6], [7], growth factor signaling and sequestration [8], degradative and modifying enzymes [9]. Matrix dysregulation is associated with severe consequences in development and disease pathogenesis, including partial bladder outlet obstruction [10]–[13]. During matrix dysregulation, many enzymes including matrix metalloproteinases (MMPs) induce denaturation of collagen, which causes exposure of cryptic epitopes. Cryptic neoepitopes found in denatured collagen but not native fibrillar collagen can induce mitogenic and hypertrophic behaviour in cells [3], [12], including smooth muscle.

Though normally phenotypically plastic, smooth muscle cells (SMC) placed into distinct matrical environments can stably retain characteristics attained on original environments [3], [14]–[16]. During disease, changes in matrix deposition and remodeling (for review see Aitken and B**ä**gli, 2009 [10]) are accompanied by a shift of SMC toward the synthetic-proliferative vs. contractile phenotype [17]–[21], as seen through decreased expression of differentiated SMC markers [22]–[31], and increased expression of growth factors [32]–[38]. The plasticity of SMC during disease is considered to diminish and become more rigid via epigenetic means [39]. This raises the possibility that matrix can exert its effects on phenotype specifically via epigenetic mechanisms.

Epigenetic change has been defined previously as ***“the structural adaptation of altered chromosomal states so as to perpetuate altered activity”*** [40] **.** Epigenetic mechanisms include histone modifications, RNA interference, and DNA methylation of CpG-dinucleotides. DNA methylation occurs on the fifth carbon of cytosine via DNA methyltransferase enzymes (DNMT1, two isoforms of DNMT3A, and several DNMT3B isoforms) [41], [42]. CpG methylation at critical regulatory regions can lead to down regulation of expression, through association with histone marks, alterations in folding of genomic DNA, and changes in accessibility of transcription factors.

Gene expression and long-term phenotypic changes observed during fibroproliferative diseases of smooth muscle have been studied in an epigenetic context [43]–[46]. Visceral SMC from neurogenic bladder possess a synthetic phenotype (producing more collagen than normal SMC). This collagen-production has been shown to be inhibited by 5′-aza-deoxycytidine, an inhibitor of DNA methylation [47]. Neurogenic compared to normal bladder SMC also proliferate at a higher rate and continue to express unique expression patterns, even after many passages [48], [49]. Both vascular and visceral SMC are quiescent on native collagen, but become highly proliferative on the mitogenic substrate of denatured collagen [3], [14]. However, when re-plated onto native collagen from denatured collagen, SMC do not easily or completely revert to the quiescent or differentiated phenotype observed if cultured first on native collagen. Together these studies suggest that epigenetic mechanisms may play a crucial role in the alteration of SMC function.

Although matrix and epigenetics are individually critical for proper smooth muscle function and phenotype, **matrix regulation** of epigenetic “machinery” or enzymes has not been clearly explored in smooth muscle cells. In other cell types, matrix components have been shown to up or down regulate enzymes involved in DNA methylation, e.g. the repressive effect of laminin substrates on DNMT1 in breast cancer cells [50].

In the present study, we explored how damaged matrix, a common stimulus in the microenvironment of hypertrophic visceral smooth muscle, alters regulation of DNA methyltransferase 3A (DNMT3A). We demonstrated that fibroproliferative stimuli exert compelling effects on the level and localization of expression of DNMT3A and DNMT3B, the enzymes most often involved in *de novo* DNA methylation responses. Upon stimulation by exposure to denatured collagen, DNMT3A signal was localized in a time, cell density, and mitosis dependent manner, through ERK-integrin cell signaling mechanisms. A stimulus common to bladder obstructive disease, hypoxia, was able to further increase the expression and localization of DNMT3A on denatured collagen. Plating human bladder SMCs on denatured matrix leads to discrete and significant changes in DNA methylation of SMC differentiation related genes, suggesting that the matrix is able to not only upregulate expression of DNMT3a but also increase DNA methylation itself.

## Results

### Damaged Matrix-induced cell proliferation and de-differentiation is dependent on DNMT activity

**Figure 1:**
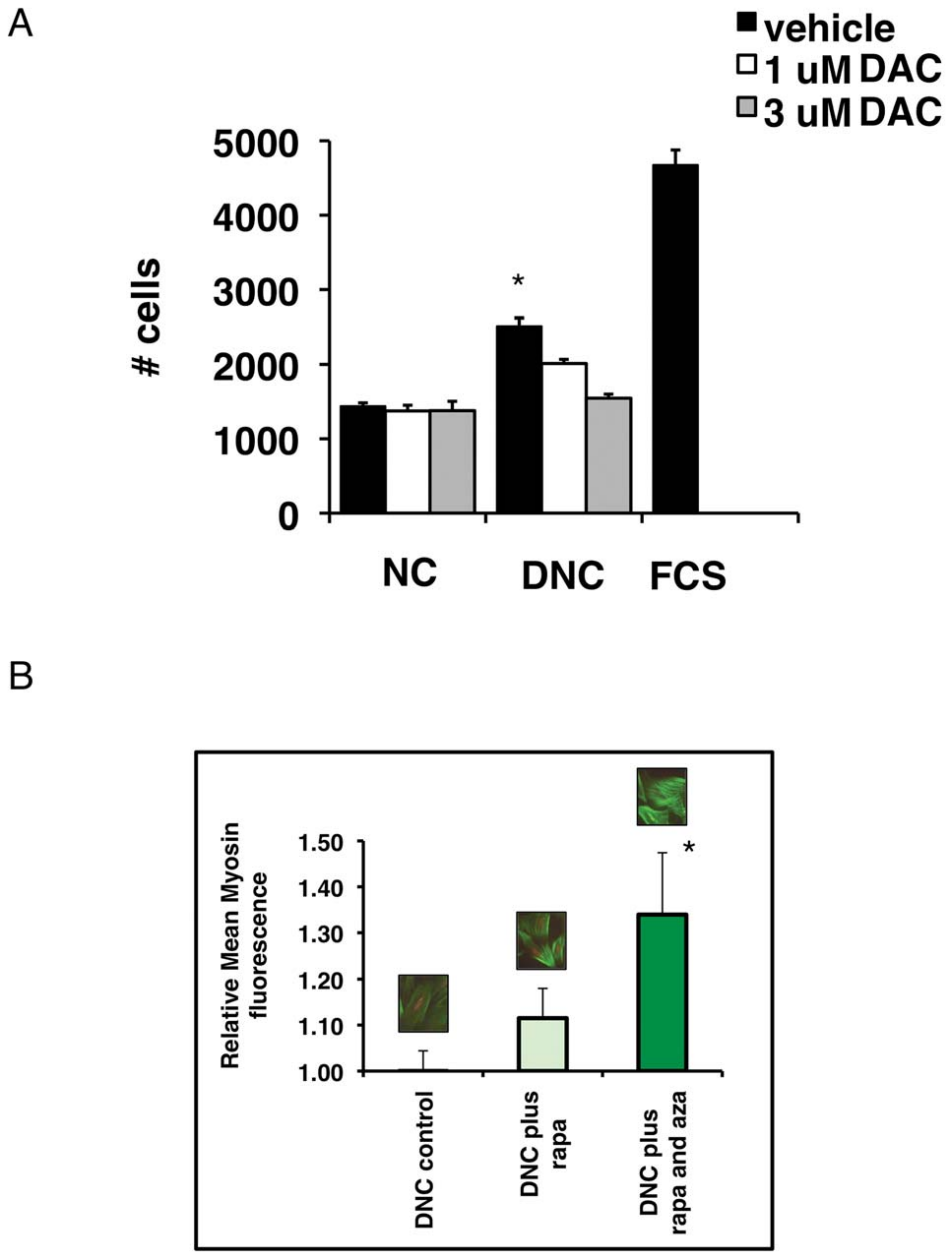
Proliferation and de-differentiation of SMC on denatured matrix depends on DNMT activity in visceral smooth muscle cells. (A) SMC were plated on native (NC) or denatured collagen (DNC) at low density (2×10^4^ cells/mL) for 6 hours in EMEM with 6% FCS, then treated with 5-aza-2′-deoxycytidine (DAC) or vehicle for another 42 hours in EMEM with 2% FCS. Six different fields per treatment for cells positive for DAPI were examined at 5× magnification and counted using Volocity analysis software, and averaged to obtain the # cells/field. * p<0.05 vs DAC treatments. (B) Loss of smooth muscle myosin could be reversed with rapamycin plus DAC. **Before treatment**, SMC were cultured for 48 hours *in vitro* on damaged collagen matrices (DNC), which suppressed expression of the differentiation marker Myosin (relative immunofluorescence expression  = 1.0). The mTOR inhibitor rapamycin alone showed only a trend in increasing Myosin expression (p = 0.11), but combined use of epigenetic inhibition (with DAC) + rapamycin significantly restored myosin expression (*p<0.04).

Previously we reported that SMC proliferation increases on damaged collagen matrix (DNC) vs native collagen (NC) matrix (Herz *et al*, 2003) [14]. Here, we tested if DNC–induced cell proliferation depends on DNMT activity, using the DNA methylation inhibitor, 5′-aza-2′-deoxycytidine (decitabine, DAC). Denatured collagen greatly stimulated proliferation, whereas native collagen rendered SMC quiescent. Treatment with decitibine after initial adherence of SMC to the matrix substrates prevented the denatured collagen-induced increase in cell numbers, while the number of cells on native collagen were unaffected by DAC (Figures 1A and S1A). The proliferative phenotype in SMC was accompanied by a loss of SMC differentiation markers by immunostaining (Figures 1B and S1B). Loss of smooth muscle myosin expression in cells plated on DNC was prevented by combined epigenetic and signaling inhibitor treatment.

After two days of culturing on damaged matrix, altered SMC myosin expression was not reversed following two days of rapamycin treatment. Previous experiments showed that rapamycin can **prevent** loss of myosin on denatured collagen. However, recovery of myosin after prior culture on damaged matrix, was only seen by combining rapamycin with epigenetic inhibitor treatment (DAC) (Figures 1B and S1B).

### Matrix alters intracellular DNA methyltransferase 3A (DNMT3A) localization and expression in visceral smooth muscle cells

**Figure 2:**
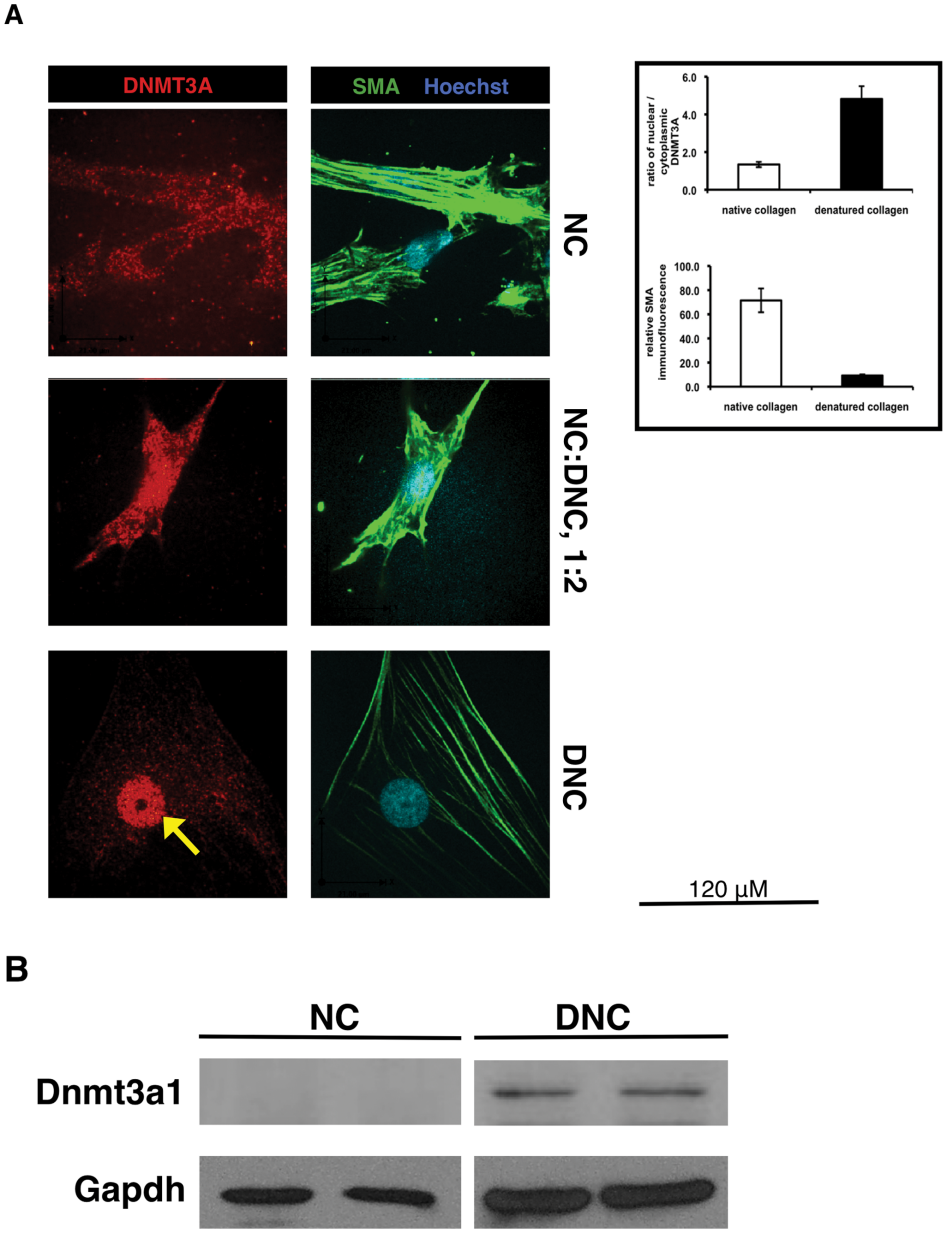
Matrix is a critical determinant of DNMT3A expression in visceral smooth muscle cells. SMC were plated on native (NC) or denatured collagen (DNC) at low density (4×10^4^ cells/mL) for 6 hours in EMEM with 6% FCS, then media was changed to 2% FCS in EMEM. (A) DNMT3A expression increases in the nucleus in response to denatured matrix, while α-smooth muscle actin (α-SMA) expression decreased. By immunofluorescent staining, levels of DNMT3A and SMA were examined with spinning disk microscopy using Volocity software, then analysed with Image J. *, p<0.05. (B) Western blotting of DNMT3A1 in protein extracts isolated from rat bSMC cultured on NC and DNC. Damaged matrix induced higher protein expression of DNMT3A1 (120 kDa).

As DNMT inhibition prevented SMC proliferation on denatured collagen, we asked if this matrix could alter DNMT3A protein or mRNA expression (Figure 2). Nuclear expression of DNMT3A was strongly increased in cells cultured on DNC in contrast to cells cultured on native collagen (Figure 2A). Increasing proportions of denatured collagen led to an increase in nuclear DNMT3A staining. In order to confirm the specificity of antibodies utilized, we transiently co-expressed DNMT3A and GFP in cells plated on DNC, and immunostained for DNMT3A. DNMT3A was found to increase with Green signal, and also did not result in overflow of the signal to the cytoplasm (Figure S2). We also examined whether DNMT3A protein expression was upregulated on DNC. Interestingly, we saw a clear increase in DNMT3A protein expression from cells plated on DNC by western blot (Figure 2B).

### Fibroproliferative Co-stimuli in DNMT expression

**Figure 3:**
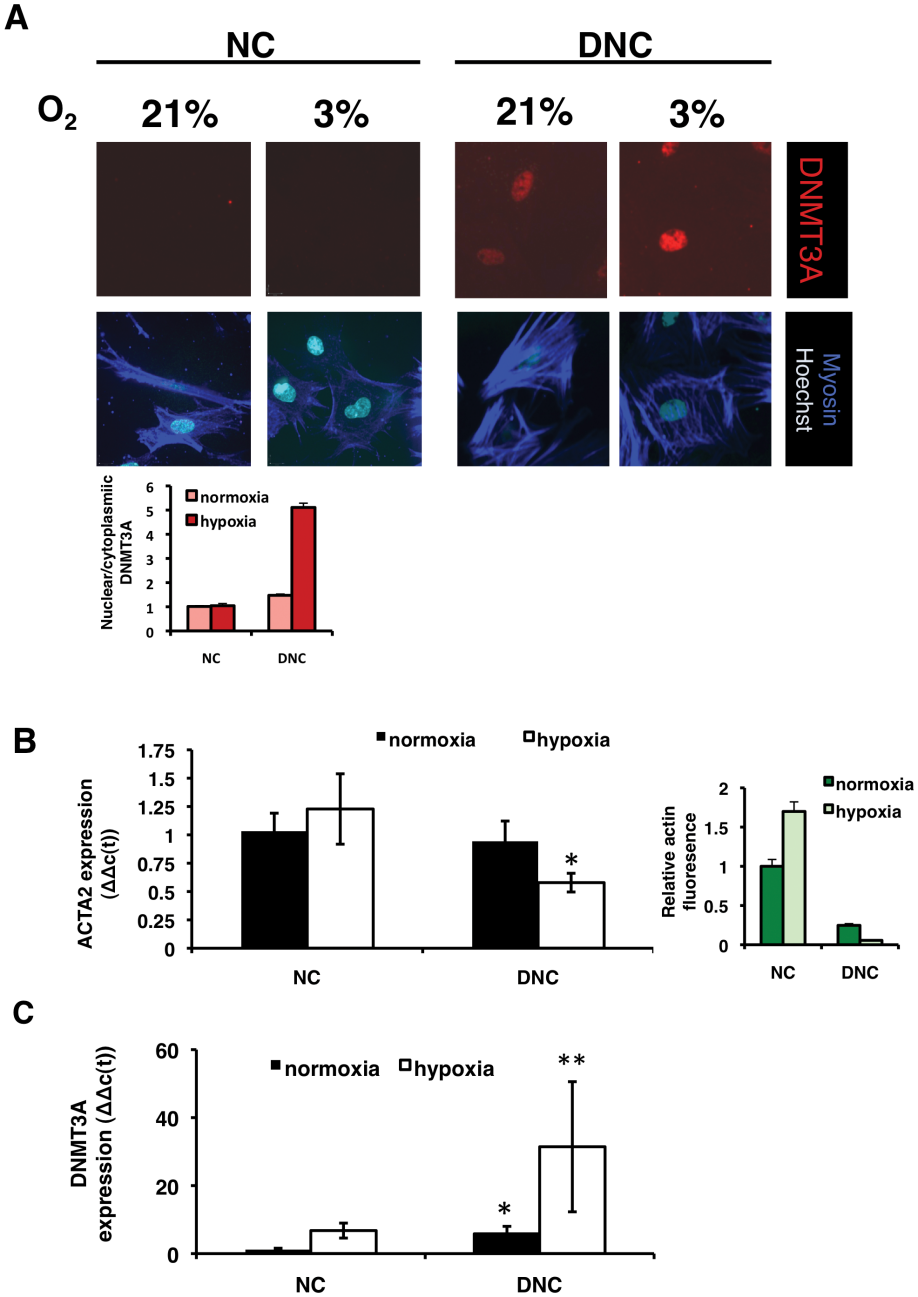
Hypoxia and damaged matrix increase DNMT3A nuclear expression in a cooperative fashion. SMCs were plated on native (NC) or denatured collagen (DNC) at low density (4×10^4^ cells/mL) for 6 hours in EMEM with 6% FCS, then media was changed to 2% FCS in EMEM. (A) SMC were plated on native (NC) or denatured collagen (DNC) and cultured under normoxia (21% O_2_) or hypoxia (3% O_2_). Hypoxia significantly enhanced the nuclear expression of DNMT3A and the down-regulation of myosin. By immunofluorescent staining, levels of DNMT3A and smooth muscle myosin heavy chain (MHC, smooth muscle-specific form) were examined by spinning disk microscopy using Volocity software, then analysed with Image J. *, p<0.05. (B) Expression of α-SMA was significantly decreased under the combined stimulation by hypoxia and damaged collagen, compared to native collagen. Both PCR and immunofluorescent staining with anti-smooth muscle actin antibody revealed a significant decrease in actin expression only on denatured collagen. (C) The expression of DNMT3A is upregulated in DNC compared to NC. Consistent with immunofluorescent staining data, the upregulation of DNMT3A mRNA expression on DNC is enhanced by hypoxia.

We asked whether hypoxia as a co-stimulus in fibroproliferative diseases [51] altered alters DNMT3A expression upon exposure to native or damaged matrix. We used parameters that induce visceral SMC proliferation [51] [52]. On DNC, hypoxia significantly enhanced the nuclear localization and expression seen on DNC alone, as well as diminished myosin expression to a negligible level (Figure 3A). SMA expression was reduced (Figure 3B), while DNMT3A mRNA expression and fluorescent signal was significantly potentiated by hypoxia on DNC (Figure 3A, C).

**Figure 4:**
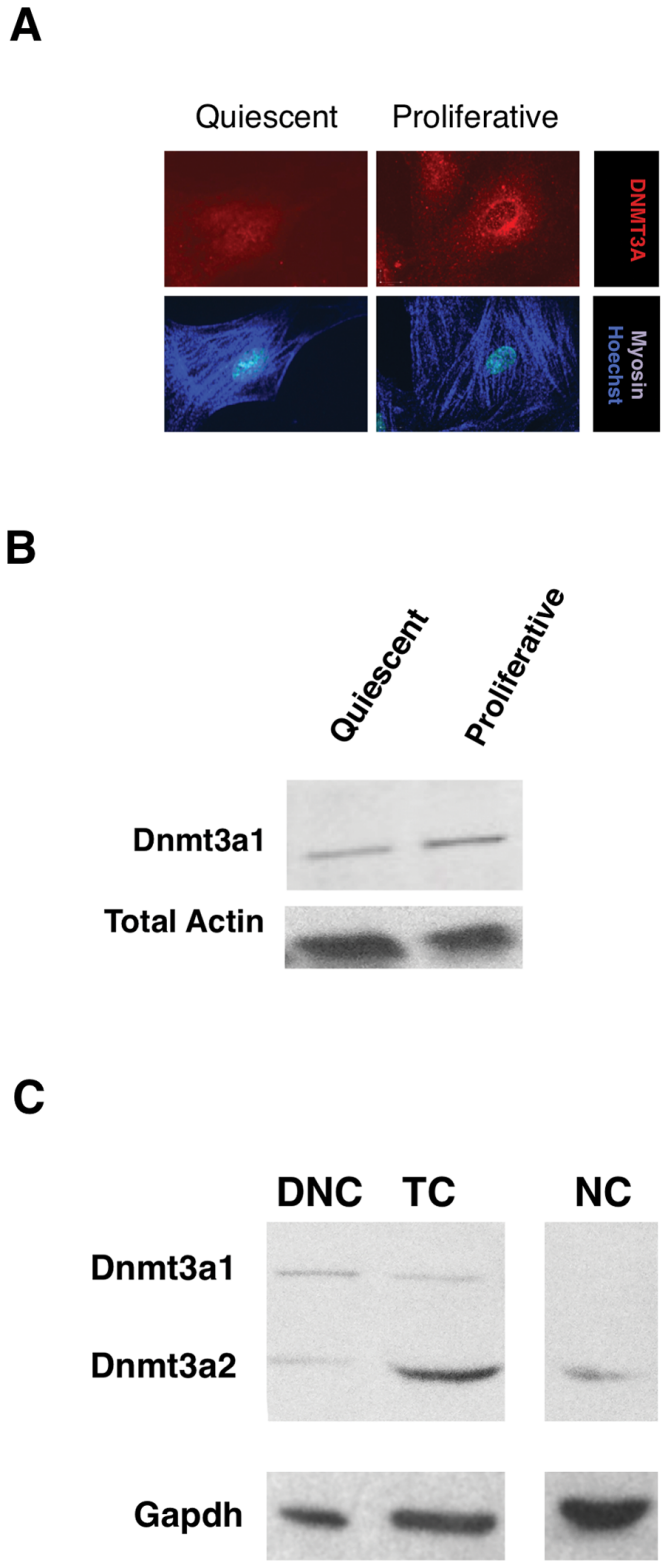
Expression of DNMT3A is differentially regulated by proliferative state on tissue culture plastic. SMC were plated as in Figure 1 then either fixed for immunstaining or harvested for protein and analysis by western. (A) Immunofluorescent staining of DNMT3A and myosin in quiescent (serum-starved) or proliferative (10% serum) SMC on tissue culture plastic (TC). Proliferative SMC express more peri-nuclear/cytosolic DNMT3a compared to quiescent SMC. (B) Western blotting of DNMT3A1 in extractions of SMC show that proliferative vs. quiescent (serum starved) SMC (plated as in Figure 4.A) expressed higher levels of DNMT3A1 protein (120 kDa) compared to quiescent SMC. (C) Western blotting of DNMT3A isoforms in SMC plated on DNC, TC and NC. DNMT3A1 shows more expression on DNC than on TC but is not expressed on NC. On the other hand, DNMT3A2 shows less expression on DNC compared to NC while showing the most protein expression on TC.

To further confirm the expression patterns seen by immunocytochemistry, we performed westerns for DNMT3A (Figure 4A–C). DNMT3A antibody stained positively in the cytoplasm and peri-nuclear region of SMC grown on tissue culture plastic in the presence of 20% serum (proliferative cells, Figure 4A). Quiescent cells in 0% serum demonstrated low levels of staining. Furthermore, serum-starved SMC (quiescent) demonstrated a lower proportion of the DNMT3A1 isoform than proliferative SMC (Figure 4B). By western blotting of nuclear and cytoplasmic extracts from cells on tissue culture plastic, we also confirmed that bladder SMC expressed both cytoplasmic and nuclear DNMT3A, with both the higher and lower molecular weight isoforms of DNMT3A (DNMT3A1 and DNMT3A2, respectively) present (Figure 4C).

### Matrix regulation of DNMT3A depends upon time after plating, transcription and translation

**Figure 5:**
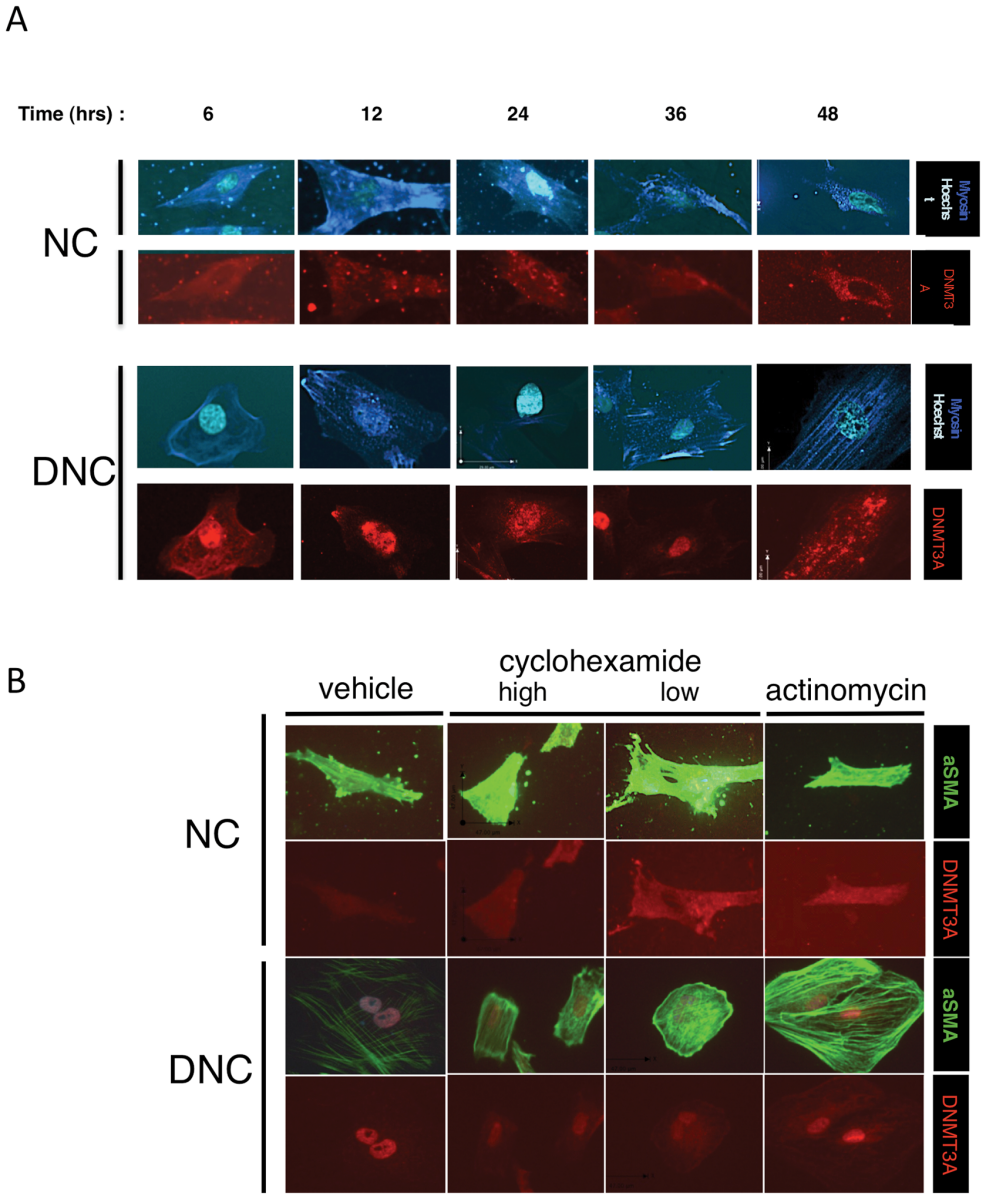
Nuclear Localization of DNMT3A is dependent upon the time after plating and transcription. (A) Timecourse of intracellular DNMT3A expression/localization after plating cells on NC and DNC. DNC plated cells show stronger DNMT3A signals overall than NC plated cells. The 36 hour timepoint shows strong signal in the nucleus of DNC plated cells. At 48 hours there continues to be high expression in the DNC cells, though the nuclear stain was not as clear as the 36 hour timepoint. NC cells did not show nuclear staining. (B) DNMT3A nuclear localization is slightly affected by inhibitors of transcription (actinomycin D) and translation (cyclohexamide) on NC, but downregulation on DNC strongly depends on both functions. SMC were plated for 4 hours as in Figure 1 and treated with cyclohexamide or actinomycin for the next 44 hours.

Since the response of cells to biological matrix microenvironments can require time and transcriptional mechanisms, we determined if DNMT3A localization to the nucleus depended upon the time after plating (following adherence and spreading), or was regulated by transcription and translation. A time course of intracellular DNMT3A expression of SMC cultured at low-density on DNC and NC matrices showed that nuclear localization occurred within 48 hours after plating, but became restricted to the nucleus after 24 hours of plating when plated at low density (Figure 5A). On NC also at low cell density, nuclear DNMT3A was observed only weakly in SMC at 6 hours after plating, when attachment and spreading is occurring, and disappeared after 12 hours. Moreover, expression of the differentiation marker SMA in cells on denatured collagen gradually decreased over time inversely with the increase in DNMT3A nuclear localization.

We examined whether transcription and translation are required for nuclear upregulation of DNMT3A using chemical inhibitors of these processes. Inhibition of transcription by cyclohexamide downregulated DNMT3A nuclear expression. Actinomycin D appeared to have only a mild, if any effect on DNMT3A localization, though it appears that both transcription and translation are completely required for the downregulation of SMA on DNC (Figure 5B).

### Matrix regulation of DNMT3A is cell density dependent

**Figure 6:**
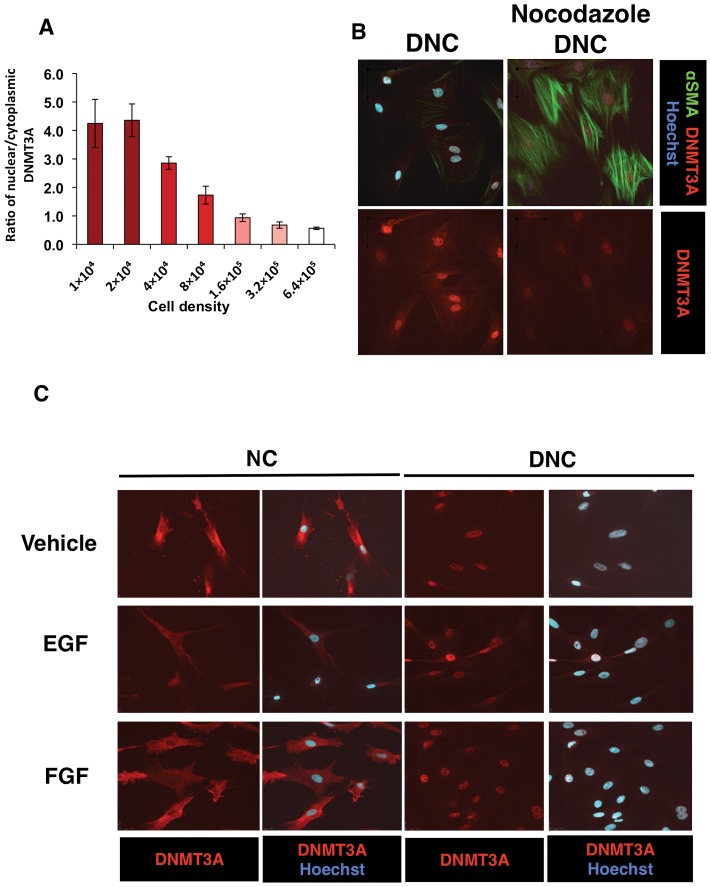
DNMT3A expression is regulated by cell-density, mitosis but not mitogenic growth factors. SMC were plated as described in Figure 1. (A) Cell density affects localization of DNMT3A to the nucleus. (B) Nuclear expression of DNMT3A is decreased by the mitotic inhibitor nocodazole in cells. (C) EGF (50 μg/mL) and FGF (10 μg/mL) fail to alter nuclear localization from patterns established on NC or DNC.

We previously reported that after two days of SMC culture at low initial density, DNC can induce a high level of mitosis [14]. As SMC on DNC were proliferating at a higher rate, we found that low cell density or mitosis is required for DNMT3A localization (Figure 6A, B). Conversely, higher cell densities were associated with a lower ratio of nuclear to cytoplasmic DNMT3A. In order to address the question of the specific role of mitosis in this response, we used the mitotic inhibitor, nocodazole, to examine if the regulatory effect of matrix remains (Figure 6B). This inhibitor was able to prevent localization of DNMT3A. Interestingly, SMC mitogens (e.g. FGF; [53]), were not able to increase DNMT3A on NC, and had no effect on DNC DNMT3A expression (Figure 6C). In response to FGF2 and EGF, the effect of the matrix predominated (Figure 6C), as neither factor altered the DNMT3A localization patterns specific to native or denatured matrix.

### Signaling pathways regulate Dnmt3a localization on damaged matrix

**Figure 7:**
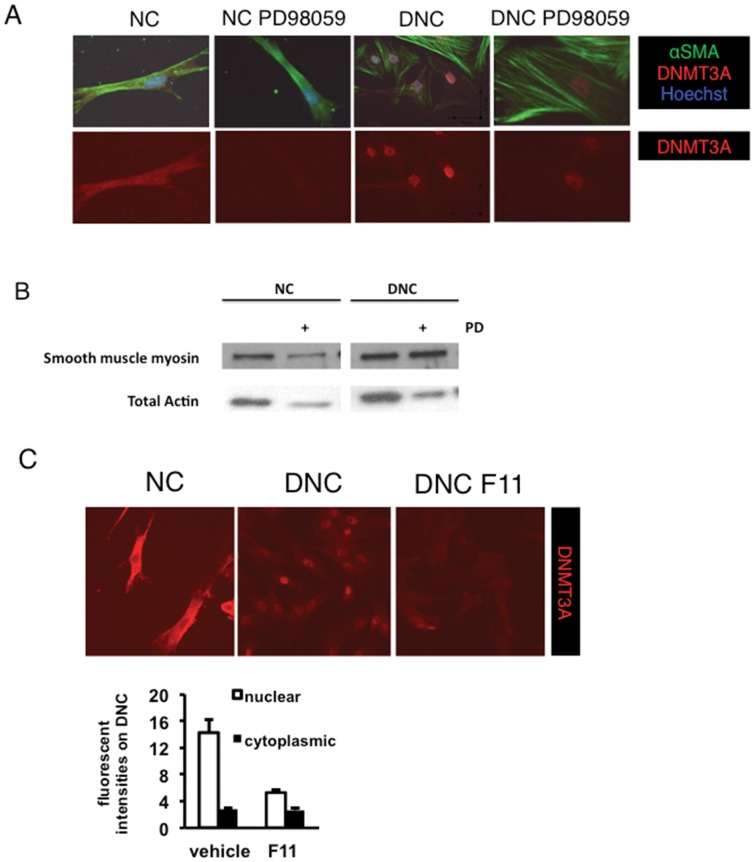
DNMT3A expression is inhibited by ERK and F11 inhibitors on DNC. The ERK integrin pathway participates in matrix induction of DNMT3A. The pathway inhibitor of ERK (40 μM PD985059) affects nuclear expression of DNMT3A, and prevents the loss of SMA and myosin expression on DNC as well as on NC (A, B). (C) DNC induction of DNMT3A nuclear localization is dependent upon integrin signaling. The blocking antibody F11, which prevents β3 integrin signaling, attenuated DNMT3A nuclear expression.

Previously, we found that proliferation of SMC in response to obstructive stimuli including damaged matrix is associated with several signaling pathways, including MEK/ERK [54] and JAK2/STAT3 [55]. We also noted that JAK2 inhibitors uncoupled epigenetic modulation of differentiation, decreasing DNMT3A localization and proliferation, but not SMA expression [55], [56]. Here, we examined how inhibition of MEK/ERK and integrin pathways by small molecule and blocking antibodies, respectively, affect DNMT3A localization in response to matrix (Figure 7A, B). It has been shown that PD98059 prevents SMC growth [51], [54] on DNC, similar to decitabine (Figure 1). We found that PD98059 decreased DNMT3A expression in both the nucleus on DNC and the cytoplasm on NC (Figure 7A). Interestingly, the previously observed coordinate downregulation of SMA and myosin expression on DNC was completely prevented by MEK inhibition (Figure 7A, B).

Integrins, including β3 integrin, play pivotal roles in SMC phenotypic switching [4], [57]. We examined the role of β3 integrin using a blocking antibody, F11 (Figure 7C). Strikingly, F11 completely abrogated DNMT3A nuclear localization in SMC on DNC.

### Matrix induces significant changes in DNA methylation

**Figure 8:**
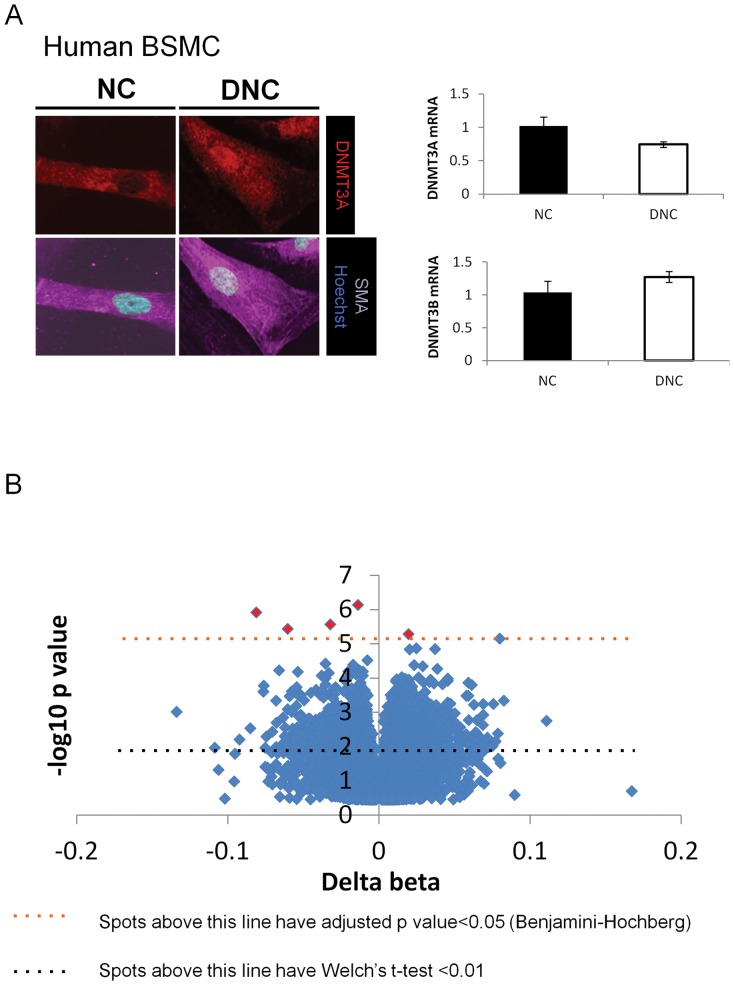
Damaged matrix induces DNMT3A nuclear expression in human bladder SMC and changes in methylation status in CpG sites of the Illumina 450K methylation array. (A) Human bladder smooth muscle cells were plated on native (NC) or denatured collagen (DNC) at low density (4×10^4^ cells/mL) for 6 hours in EMEM with 6% FCS, then media was changed to 2% FCS in EMEM. Nuclear expression of DNMT3A is increased in SMC cultured on DNC. By immunofluorescent staining, levels of DNMT3A and smooth muscle myosin heavy chain (MHC, smooth muscle-specific form) were examined by spinning disk microscopy using Volocity software, then analysed with Image J. DNMT3A and 3B were both examined by QPCR. While DNMT3A levels were not significantly increased by mRNA expression, protein expression of DNMT3A and DNMT3B levels were increased *, p<0.05. (B) Illumina 450 K CpG methylation array of human SMC plated onto NC and DNC show several significant changes at discrete hypomethylated and hypermethylated CpG sites on DNC compared to NC (see Table S2 for raw data). Red diamonds indicate significantly altered CpG methylation (adjusted p<0.05, by Benjamini-Hochberg).

Damaged matrix is a persistent stimulus to bladder smooth muscle cells caused by bladder obstruction *in vivo.* In order to examine DNA methylation events associated with a damaged collagen matrix, we took a genome-wide approach using the Illumina 450 K methylation array to probe bisulfite-converted DNA from human bladder smooth muscle cells. We first, however, confirmed that human BSMC showed a similar pattern of DNMT3A localization as rat BSMC on DNC vs. NC at 48 hours (Figure 8A). DNMT3A and 3B mRNA expression was either stable or increased in the human BSMCs (Figure 8B).

The DNA methylation array data was analysed first by comparing methylation between the groups from the two substrates using pre-filtering for a significant Welch's t-test, and secondary correction for multiple testing by Benjamini-Hochberg (see Table S2 for raw data). The overall data distribution showed minimal changes and a similar amount of hyper and hypo-methylation, with only a small number of sites showing significant changes after correction for multiple testing (Figure 8B). Damaged matrix induced several significant and discrete alterations in CpG methylation in primary human smooth muscle cells within a relatively short amount of exposure time (2 days) (Table 1). When specific genomic regions were analysed, several sites appeared to be significantly altered, including five in exon1 regions and three in 5′-UTRs of several genes (Table 1), while the overall distribution of the genes with a t-test<0.01 were in CpG Islands with few changes in differentially methylated regions (DMRs) (Figure S3).

**Table 1:** Differentially methylated CpG sites (T-test <0.01, adjusted p value <0.05, Benjamini-Hochberg) revealed after analysis of specific regions or all sites of the epigenome.

Genomic Region Analysed	Gene closest to CpG site	P-Value	Adjust Pval	Beta-Difference	Mean Beta DNC	Mean Beta NC
Total Genome	TUSC3; TUSC3	0.000000734	0.02251	−0.0138	0.084	0.098
Total Genome	HLA-DQA2	0.00000362	0.03355	−0.0603	0.367	0.427
Total Genome	C7orf4	0.00000271	0.03355	−0.0323	0.219	0.251
Total Genome	RSP14 (Island)	0.00000519	0.03849	0.0195	0.047	0.027
Total Genome	RCOR2 (Island)	0.00000689	0.04264	0.0798	0.398	0.318
Transcription start site 1500 bp upstream	LOC100303749	0.000000105	0.00214	−0.0273	0.862	0.889
5'UTR	CLTCL1	0.000002524	0.03571	0.0293	0.282	0.253
5'UTR	AP1S1	0.000010467	0.04936	0.0756	0.323	0.247
5'UTR	GORASP2	0.000007544	0.04936	0.0378	0.314	0.276
Exon 1	SLC5A9	0.000000948	0.01478	−0.0219	0.459	0.48
Exon 1	CLTCL1	0.000003128	0.02438	0.0293	0.282	0.253
Exon 1	AP1S1	0.000012526	0.03983	0.0756	0.323	0.247
Exon 1	GORASP2	0.000009119	0.03983	0.0378	0.314	0.276
Exon 1	LYZ	0.000012776	0.03983	−0.0274	0.605	0.632
gene body	DEDD	0.000000665	0.01269	−0.0243	0.762	0.786
3'UTR	C8orf44	0.000000835	0.01092	−0.0443	0.81	0.854
Island	chr13:114912875-114913457	0.000000075	0.00201	−0.0245	0.721	0.746
Shelves and shores not significantly altered						

Highlighted genes appear more than once in the Table.

**Figure 9:**
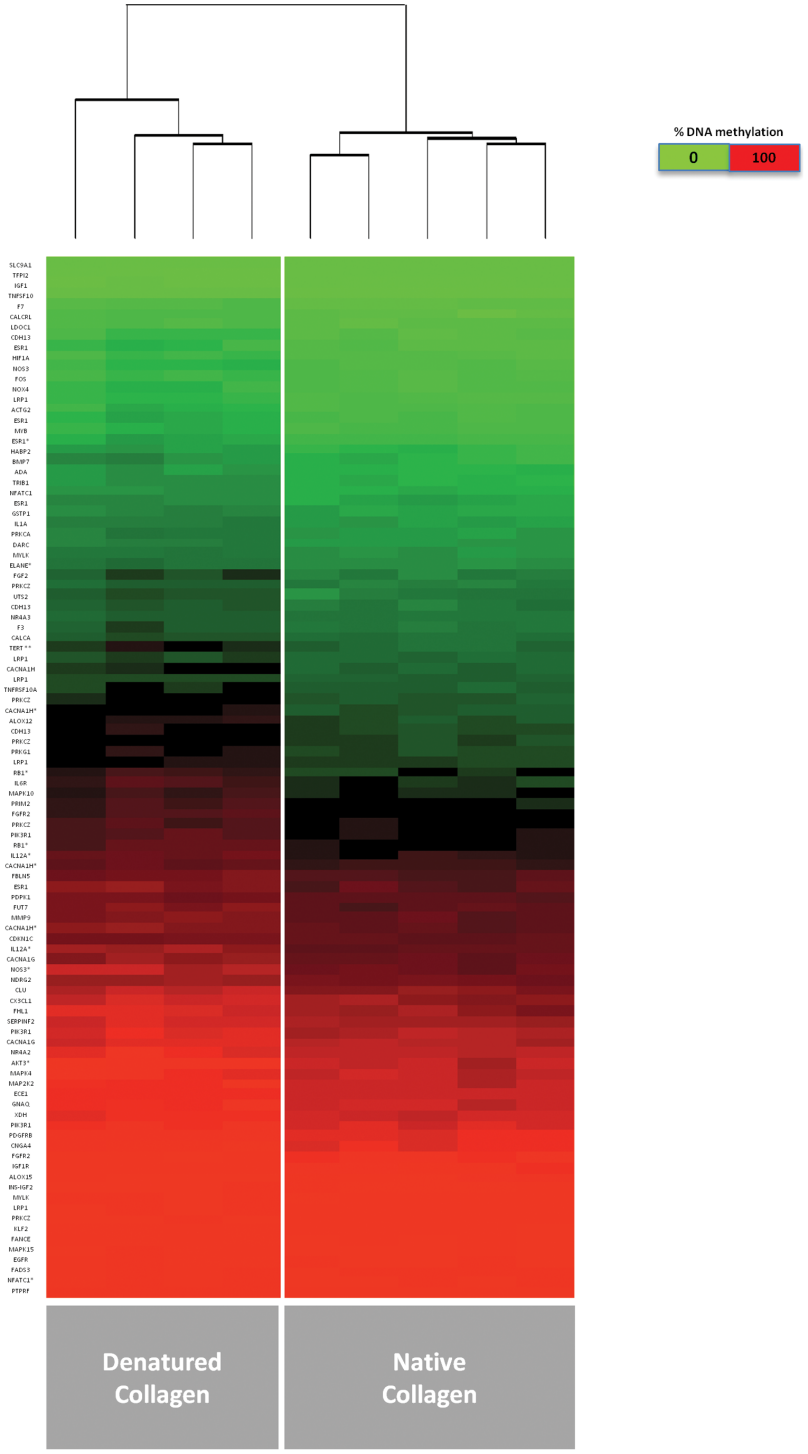
*A priori* test of CpG sites in SMC specific genes reveals a trend towards hypermethylation. A priori test was performed to select 6831 CpG sites associated with SMC specific genes from the Illumina 450 K array data, followed by t-test and Benjamini-Hochberg correction for multiple testing. Heat map demonstrates a global shift in hypermethylation in top 100 SMC-related CpG sites.

**Figure 10:**
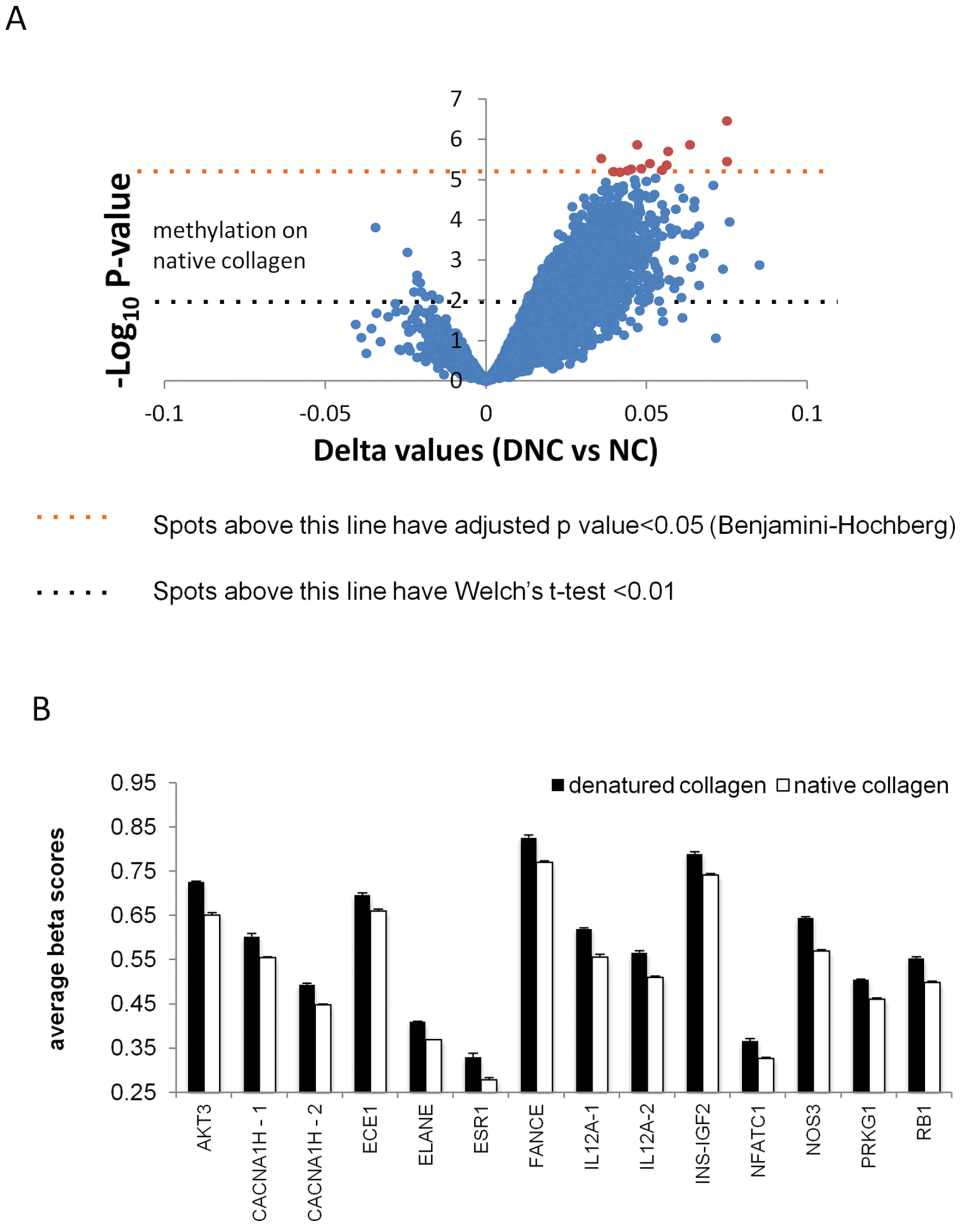
*A priori* test of CpG sites in SMC specific genes reveals specific changes in DNA methylation. (A) Volcano plot of hypomethylated and hypermethylated CpG sites reveals a clear trend toward hypermethylation of sites in cells plated on DNC. 14 CpG sites have statistically significant increase in methylation. (B) Beta values (degree of methylation) in 14 CpG sites near 12 genes differed between cells cultured on NC and DNC. Differences between cells on native collagen and denatured collagen were significantly altered in all sites (adjusted p value <0.05).

We then performed an *a priori* analysis of only the CpG sites linked to SMC differentiation related genes (Table S1). After correction for multiple testing, we found a higher level of overall hypermethylation of SMC genes in cells on DNC vs. NC (Figures 9 and 10A), and 14 significantly altered sites (Figure 10B) proximal to 12 genes, after correction for multiple testing. While the changes in these significantly altered sites may appear modest compared to the massive dysregulation seen in cancer, the errors within each group in these significantly altered genes were very small and consistent (Figure 10B), and may represent characteristic epigenetic signatures of benign (non-malignant) disease.

## Discussion

### Summary of main findings

The present study examines how stimuli, known to induce proliferative, synthetic and hypertrophic phenotypes in SMC, particularly in the context of hollow organ obstruction, alter expression of SMC DNA methylation machinery only on damaged matrix. While matrix has long been considered an important player in cell phenotype [3], [4], [58]–[62], this is the first report to show that matrix alterations can incite changes in DNA methylation machinery and DNA methylation in SMC. We show that the *de novo* methylator DNMT3A is an active component in the dynamic shift in visceral SMC phenotypic states in response to stimuli coordinately occurring during organ obstruction. Furthermore, we have shown that matrix has a significant effect on a discrete set of DNA methylation sites.

### DNMT isoforms on different matrices

We have confirmed by Western analysis that visceral SMC express two major DNMT3A isoforms (Figure 3). These two isoforms, include the full-length DNMT3A and a shorter but still functional DNMT3A2 with an exon internal to an intron in the full-length DNMT3A. DNMT3A2 is found in the cytoplasm and the nucleus where it usually co-localizes with euchromatin (consisting of mainly single copy genes which are often transcribed). Conversely, full-length DNMT3A is restricted to the nucleus, and often co-localizes with heterochromatin [63]–[65].

### Control of DNMT localization

DNMTs (1, 3A, 3B) have nuclear localization signals (NLS) in their N-terminus, but as with other NLS-containing proteins, such as c-ABL, HMGB1, CD44, AR and RB, acetylation of lysines in their NLS might lead to altered translocation by nuclear import proteins [66]–[69], thereby altering their cytoplasmic retention. There are several reports of cytoplasmic retention of DNMTs [63], both *in vivo* and *in vitro* [70]. Normal import of DNMT3A occurs via alpha-importins, which also has an NLS under control of acetylases [71]–[73]. Under homeostatic conditions, tissues could be expected to harbour a normally developed and functioning collagen matrix where DNMT3A is largely cytoplasmic. Based on the present observations, it is important to note that previous *in vitro* DNMT immunocytochemistry and western studies have used tissue culture plastic as a substrate, which usually enhances nuclear localization, and might thereby preclude the accurate and relevant detection of cytoplasmic DNMTs.

The effect of matrix and tissue culture plastic may also be cell type/line specific, as the majority of DNMT localization work has utilized cancer cell lines and embryonic stem cells, rather than normal primary cells. Indeed, in our own work, cytoplasmic DNMT1 was observed in urothelial cell lines on tissue culture plastic [74]. When inoculated with uropathogenic bacteria but not non-pathogenic bacteria, these cell lines then showed expression of DNMT1 in the nucleus. The localization pattern of DNMT3A staining and western blotting in the present study suggests that the DNMT3A2 isoform localizes in the cytoplasm in cells on native collagen. DNMT3A2 appears to function during differentiation in ES cells [75], constitutively in several organs, and during ontogeny causing DNA methylation of tissue-specific genes [76], [77]. DNMT3A1 is found at low levels in most cells, and is more important for heterochromatic CpG methylation [78]. It is intriguing then that we observe DNMT3A localizing in the nucleus in SMC undergoing bidirectional shifts in phenotypic differentiation state.

Proteasomal degradation of nuclear proteins including DNMTs is controlled by post-translational modifications, including phosphorylation by GSK3β and ubiquitinylation by the proteosomal pathway [79]–[82]. The degradative process could decrease the pool of DNMTs available for nuclear localization, as DNMTs would be retained and eventually degraded in the cytoplasm. We are presently examining the post-translational modifications on DNMTs to evaluate their contributions to DNMT3A cytoplasmic retention. In pathological circumstances, nuclear export and import mechanisms may be altered. For example, CRM1 exports HDAC1 to the kinesin motors of the cytoplasm, blocking motor activity [83]. This opens the possibility that HDACs or other molecules associate with and shuttle DNMT to different cellular compartments.

### MMP remodeling during fibroproliferative disease

*In vivo,* fibroproliferative stimuli are inextricably linked in hollow organs (e.g. bladder, heart, and vasculature), with mechanical strain inducing the expression and activation of matrix metalloproteinases (MMPs), particularly the gelatinases (MMP2 & MMP9), which can profoundly alter the matrix microenvironment [54], [84], [85]. Matrix on its own is known to exert ongoing effects on SMC phenotype due to the slow turnover of matrix components [86]. In addition, mechanical strain and pressure lead to microvascular compression in hollow organs, and consequent hypoxia [87], [88]. Hypoxia itself induces MMP7 expression and alters matrix properties as well [54]. In this context, it is interesting to note that hypoxia only enhances DNMT3A nuclear expression on damaged matrix, but is unable to induce nuclear expression on its own on NC. Unlike our previous work [51] where rapamycin could prevent de-differentiation when given immediately after cell attachment [51], we saw here that rapamycin was not able to reverse hyperplasia and loss of differentiation unless decitabine was also present. The combination of the two treatments allowed the cells to recover myosin expression and to slow their growth (Figure S1B).

On DNC alone, the localization of DNMT3A is dependent on cell density. We speculate that this might relate to either the level of mitosis in the cells, the degree of paracrine/autocrine signaling, or cell-cell contacts. At higher densities, cells may decrease mitotic activity or increase their autocrine signaling and cell-cell contacts.

### Epigenetics of fibroproliferation

The importance of epigenetics in SMC disease is only beginning to be appreciated with few studies published in the literature. The one area of epigenetics that has been reported consistently is histone modifications in SMC marker genes (e.g. SMA, SM-MyHC, calponin and caldesmon) [44], [89]. In contrast to epigenetic studies of fibroblasts [90], [91] [92]–[100], relatively few papers have examined DNA methylation and methylation machinery in SMC. Expression of collagen type I and III genes in visceral SMC are regulated in part by DNA methylation [47]. In one PCR array study using a false discovery rate <1, a *in vitro* model of SMC differentiation was somewhat associated with downregulation of DNMT expression [101]. Similarly, PDGF-induced mitogenic activity in SMC is dependent upon epigenetic mechanisms [89], [102]. Conversely, embryonic differentiation of smooth muscle is associated with increased DNMT expression. In the present study, DNC with and without hypoxia increases DNMT3A localization and decreases SMA expression. It will be important to understand how the context of different inciting stimuli alters the regulation of DNMT3A along with its histone and transcriptional co-factors. DNA methylation of some sites may be beneficial, while methylation of others is detrimental in these contexts. Nonetheless, the crucial role of matrix in all of the contexts examined here suggests that matrix is a crucial component for upregulation of the DNA methylation machinery in non-malignant cells [57].

### Matrix alters DNA methylation in SMC

Matrix can rapidly alter methylation of distinct CpG sites, as our array experiment duration was **only two days**. Despite known limitations with CpG array technology [103], the changes in β values at various sites after a short exposure time to matrix suggests that the extracellular matrix environment may, in part, exert its effects on regulation of gene expression through alterations in DNA methylation [104]. The number of differentially methylated CpG sites is within the range of changes seen in other MethylArray comparisons, such as dilated cardiomyopathy and end-stage heart disease [105], [106]. In contrast, in one study by Sandoval et al, 2011 [107], the comparison of colon cancer cells with 2 different normal colonic mucosae yielded only 3–6% of sites with differential DNA methylation. While the latter study compared cancer cells and normal tissue, our work examined differences in methylation in one primary cell line plated in two different environmental conditions over a relatively short period of time. In contrast to cancer cell lines or tumour tissues, the cardiomyopathy studies revealed only very discrete changes, with the majority of sites failing to show any dysregulation using standard statistical methodologies. The heart studies and our own utilize non-cancer cells or tissues, which, unlike cancer cells, still retain many of the epigenetic controls for cell differentiation. In this context then, it is actually quite striking to observe discrete alterations at 14 SMC differentiation related sites over the course of only 48 hours.

### Future studies and conclusions

In syngeneic animal models of obstruction of the urinary bladder, the level of recovery following de-obstruction can vary, depending on the prevailing microenvironmental stimuli that persist. It would be of interest to uncover predictors for the degree of recovery in a clinical setting. By examining how SMC *in vitro* respond to matrix to cause long-term changes, our goal is to identify therapeutic targets and biomarkers for intractable disease through an examination of DNA methylation patterns. Furthermore, understanding how matrix can alter DNMT expression and localization may be crucial for our understanding of the epigenetic instigators underpinning many diseases.

## Materials and Methods

### Cell Culture

Neonatal pups (postnatal days 1–3) were housed under normal light/dark conditions with their dam (with free access to food and water) until removed from the cage and sacrificed by decapitation in accordance with an approved protocol with the Animal Care Committee of the Hospital for Sick Children. Bladder domes were harvested, minced and collagenase digested for 5 minutes. Suspended cells were removed from the final digest, and tissue was further digested for another 40 minutes. Cells were grown at a density of 2 bladders/plate for approximately 1–2 weeks in 10% fetal bovine serum (Multicell) in EMEM plus antibiotic/antimycotic (Multicell), and subcultured using trypsin/EDTA (Multicell) for up to 2 passages. Cells were pre-starved for Collagen gel experiments in 0% FCS EMEM for 24–48 hours or plated and then starved before treatment for other experiments.

### Collagen Substrates

Two substrates of collagen were prepared: native collagen gels (NC) and denatured collagen (DNC). NC gels were made from type I collagen (Elastin Products Company, Owensville, Missouri, USA) at 3 mg/mL by mixing equal volumes of 6mg.mL collagen and a 0.1 M NaOH+ 2XPBS solution. NC gels were polymerized at 37°C for 1 hour. Collagen for DNC was first boiled for 20 minutes before neutralization and plating as with native collagen. All gels were washed three times in EMEM before plating cells. BSMC were added at 2×10^4^ cells/mL unless otherwise indicated in figure legends. For Figures 1 and 2, gels were plated on NC, NC mixed 1∶1 or 1∶2 with DNC, or DNC alone.

### Hypoxia

BSMCs were plated at 5×10^4^ cells/mL onto NC/DNC gel and serum-starved, before placing into the hypoxia chamber with 3.0% O_2_, 5% CO_2_ and N_2_ balance for 48 hours [51], [108].

### Immunostaining and Confocal Microscopy

Immunofluorescent staining was performed as described previously [74]. Cells were fixed in 4% PFA for 20 minutes (followed by PBS wash), followed by permeabilization in 0.2% Triton X-100 in PBS, washing, and blocking in 5% normal Goat Serum [74]. Staining was performed using mouse monoclonal anti-DNMT3A (Abcam) and rabbit polyclonal anti-smooth muscle actin (1∶200, Abcam) or rabbit polyclonal anti-myosin heavy chain (non-reactive with non-muscle forms of myosin, 1∶200, Abcam). The antibodies against DNMT3A and 3B detect most isoforms of the proteins. Secondary antibodies diluted to 1∶200 included anti-mouse-Cy3 and anti-rabbit-DyLight 488/or Far-Red (Jackson Immunolabs). Nuclear counterstaining was performed with Hoechst.

### Cell Counting

Cell counting was chosen for evaluation of the total number of cells (both proliferating and surviving). For collagen gel experiments, BrdU staining was precluded, as even fixed matrix is solubilized by the hydrochloric acid treatment step involved in this staining protocol. Cells were counted using ImageJ on a minimum of 9 fields taken at 200X original magnification, as described elsewhere [51].

### Protein extractions and Western blotting

Protein from 1×10^6^ BSMC were extracted using with 0.2%TritonX-100 or 0.5% deoxycholate in Tris buffer plus protease inhibitors (Invitrogen). After quantifying protein (BioRad protein assay), 20 μg of sample for each well were placed in Laemmli sample Buffer and denatured. Protein was electrophoresed on an 8% PAGE gel, and transferred to nitrocellulose membranes via electroblotting as described previously [55]. Membranes were blocked in BSA and skim milk powder in TBST, then incubated with antibodies against DNMT3A (abcam; 1∶1000), which detects both high and low molecular weight forms of DNMT3A, or myosin (Biomedical Labs), gapdh (Cell Signaling), actin (Sigma) or smooth muscle actin (Abcam) overnight at 4°C with shaking. Secondary anti-mouse- or anti-rabbit-HRP (1∶2000) and ECL-Plus were used to detect bands via autoradiography.

### PCR for DNMT3A

RNA from BSMC was isolated using Trizol reagent (Invitrogen), and 0.2 μg of RNA was reverse transcribed using Superscript III, as described [51]. Sequences of DNMT3A primers were taken from Lees-Murdock *et al*, 2005 [64], and were specific to *Dnmt3a (isoform a or Dnmt3a1)*. DNMT3B, gapdh and rpl19 primers were generated on Primer 3. PCR was performed on the Peltier Thermal Cycler-200 (MJ Research) with the iQ SyBR Green mix from BioRad, at 61.2°C. Quantification was performed by normalization to rpl19 or gapdh by the ΔΔc(t) method.

### Drugs and treatments

Drugs and growth factors were added after 3–6 hours after plating on collagen gels, in order to prevent interference with cell attachment. Dosages for each treatment were as follows: Nocodazole at 0.04 μg/mL (Sigma-Aldrich), 10 μg/mL cyclohexamide (LC Laboratories), PD98059 at 40 μM (Sigma), F11 antibody at 0.03 μM, (Sigma-Aldrich), FGF2 at 10 μg/mL (BD Transduction), EGF at 50 μg/mL (BD Transduction), DAC at 0.2, 1 or 3 μM as indicated (Sigma-Aldrich) and rapamycin at 5 ng/mL (LC Laboratories).

### Illumina Bead-chip analysis of DNA methylation on damaged matrix

Primary culture human bladder smooth muscle cells (obtained from PromoCell), were cultured on normal collagen and denatured collagen for 48 hours *in vitro*. DNA was extracted and bisulfite converted using the EZ DNA Methylation-Gold kit (Zymo Research), and then amplified by Illumina Infinium HD Methylation assay and hybridized to a Human 450 K methylation v1 Beadchip. The Beadchip was scanned using iScan (Illumina) and quantified in GenomeStudio Version 2011.1 (Illumina). Microarray service was provided by the University Health Network Princess Margaret Genomics Centre (www.pmgenomics.ca Toronto, Canada).

### Statistics

Comparisons between groups (with sample size always greater than 3) were performed using an analysis of variance with a *post-hoc* two-tailed t-test. A p value less than 0.05 was considered significant. For MethylArray data, statistical analysis was performed on R bioconductor IMA and Methylumi packages, for both Welch's t-test and adjusted p values. For the total epigenomic analysis, a Benjamini-Hochberg correction for multiple testing was performed on data, p<0.05 and t-test less than 0.01. For the *a priori* analysis, we analysed CpG sites proximal to SMC differentiation related genes (as identified on Ingenuity pathway analysis, See Table S1 for list of SMC genes). This set of CpG sites (which included 6,831 sites) were analysed separately from the main list, using Benjamini-Hochberg to correct for multiple testing, after which p<0.05 was considered significant.

## Supporting Information

Figure S1 **DNMT3A overexpression in bladder smooth muscle leads to nuclear expression on DNC.** GFP and DNMT3A plasmid clones (from Addgene) were overexpressed in primary bladder smooth muscle cells as described previously [51]. DNMT3A did not localize to the cytoplasm when overexpressed in cells plated on DNC, though increased expression of DNMT3A.
(TIFF)

Figure S2 **To confirm that our antibodies were specific, we stained BSMC co-transfected with GFP and DNMT3A clone (Addgene) using LTX and Plus reagent (Invitrogen), then immunostained with DNMT3A antibodies (abcam) and anti-GFP (A).** We also confirmed that the known inhibitor of DNMT3A expression, DAC in combination with rapamycin was able to downregulate DNMT3A expression (B). Cells in (B) were prepared as in Figure 1B. Cells in (A) were prepared as in Aitken *et al*, 2010[51].
(TIFF)

Figure S3 **A. Genomic Distribution of differentially methylated genes on damaged collagen vs native collagen.** 450K genes were normalized then filtered for p<0.01 by t-test. Epigenomic location was identified through annotations provided from Illlumina. Significant genes after adjustment for multiple testing (p<0.05) are listed in Table 1. **B. Distribution of differentially methylated CpG sites in DMR, CDMR and RDMR's as per Illumina's annotations for the sites.**
(TIFF)

Table S1 **List of SMC differentiation related genes.**
(XLSX)

Table S2 **Illumina 450K methylation array data.**
(XLSB)
