# Correction: Phenotypic Switching Induced by Damaged Matrix Is Associated with DNA Methyltransferase 3A (DNMT3A) Activity and Nuclear Localization in Smooth Muscle Cells (SMC)

**DOI:** 10.1371/annotation/c825c71c-bb8a-456f-9ed4-83969a76429c

**Published:** 2013-10-28

**Authors:** Jia-Xin Jiang, Karen J. Aitken, Chris Sotiropoulos, Tyler Kirwan, Trupti Panchal, Nicole Zhang, Shuye Pu, Shoshana Wodak, Cornelia Tolg, Darius J. Bägli

The name of the third author should be: Chris Sotiropoulos.

The correct citation is: Jiang J-X, Aitken KJ, Sotiropoulos C, Kirwan T, Panchal T, et al. (2013) Phenotypic Switching Induced by Damaged Matrix Is Associated with DNA Methyltransferase 3A (DNMT3A) Activity and Nuclear Localization in Smooth Muscle Cells (SMC). PLoS ONE 8(8): e69089. doi:10.1371/journal.pone.0069089 

