# Supplementary figures and images for: Phenotypic Switching Induced by Damaged Matrix Is Associated with DNA Methyltransferase 3A (DNMT3A) Activity and Nuclear Localization in Smooth Muscle Cells (SMC)
Source: PLoS One. 2013 Aug 7;8(8):e69089. doi: 10.1371/journal.pone.0069089 (PMC3735580; doi:10.1371/journal.pone.0069089)

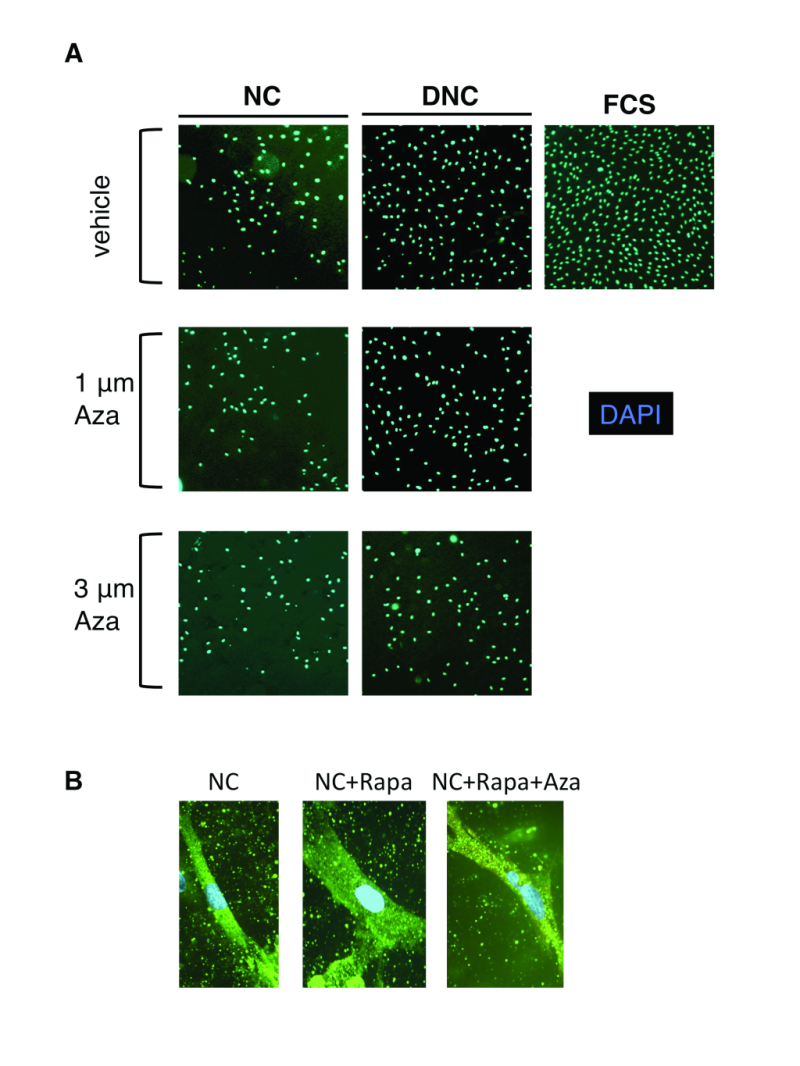

Supplement: Figure S1 — DNMT3A overexpression in bladder smooth muscle leads to nuclear expression on DNC. GFP and DNMT3A plasmid clones (from Addgene) were overexpressed in primary bladder smooth muscle cells as described previously [51]. DNMT3A did not localize to the cytoplasm when overexpressed in cells plated on DNC, though increased expression of DNMT3A. (TIFF) [file pone.0069089.s001.tif]

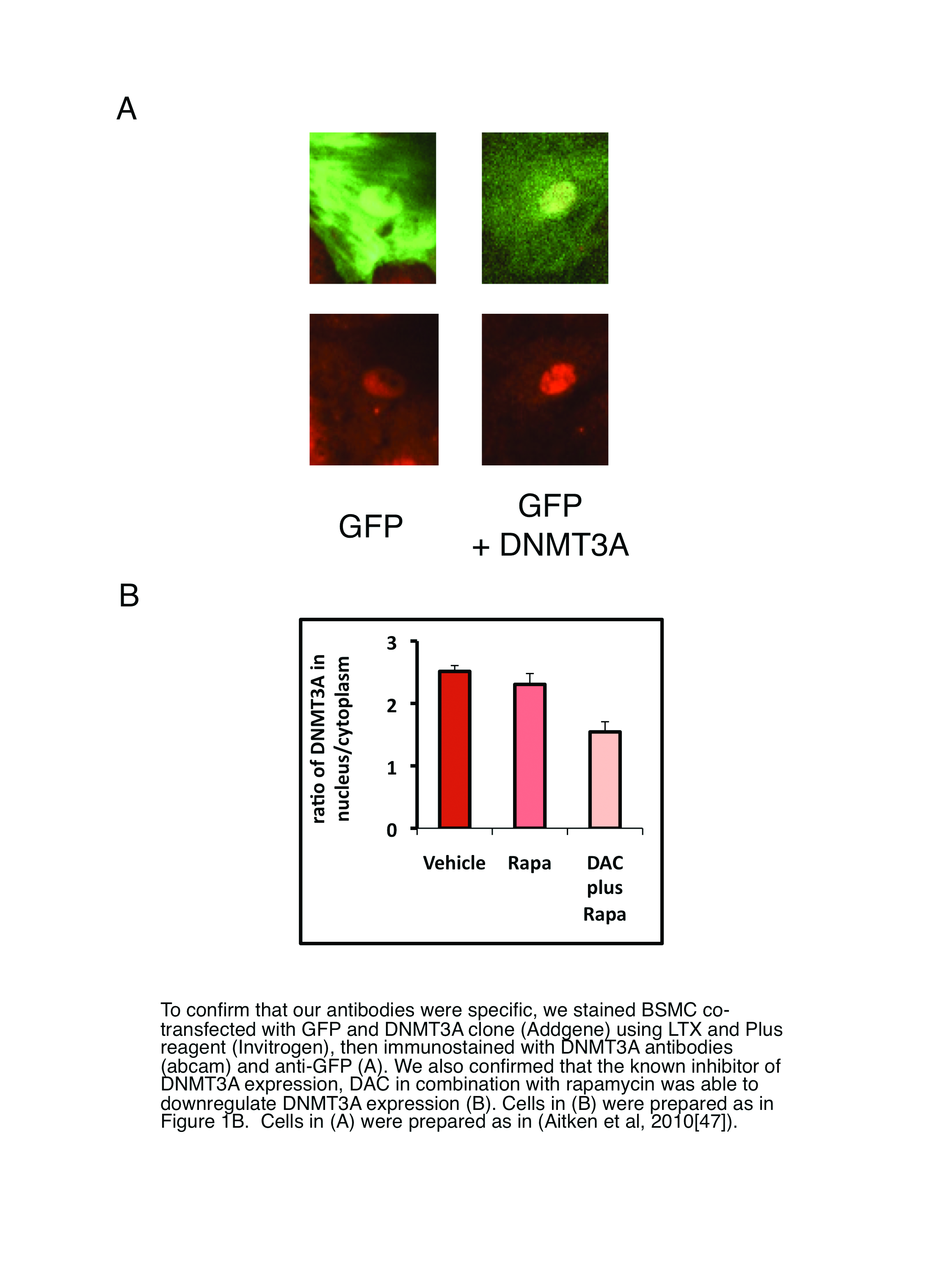

Supplement: Figure S2 — To confirm that our antibodies were specific, we stained BSMC co-transfected with GFP and DNMT3A clone (Addgene) using LTX and Plus reagent (Invitrogen), then immunostained with DNMT3A antibodies (abcam) and anti-GFP (A). We also confirmed that the known inhibitor of DNMT3A expression, DAC in combination with rapamycin was able to downregulate DNMT3A expression (B). Cells in (B) were prepared as in Figure 1B. Cells in (A) were prepared as in Aitken et al, 2010[51]. (TIFF) [file pone.0069089.s002.tiff]

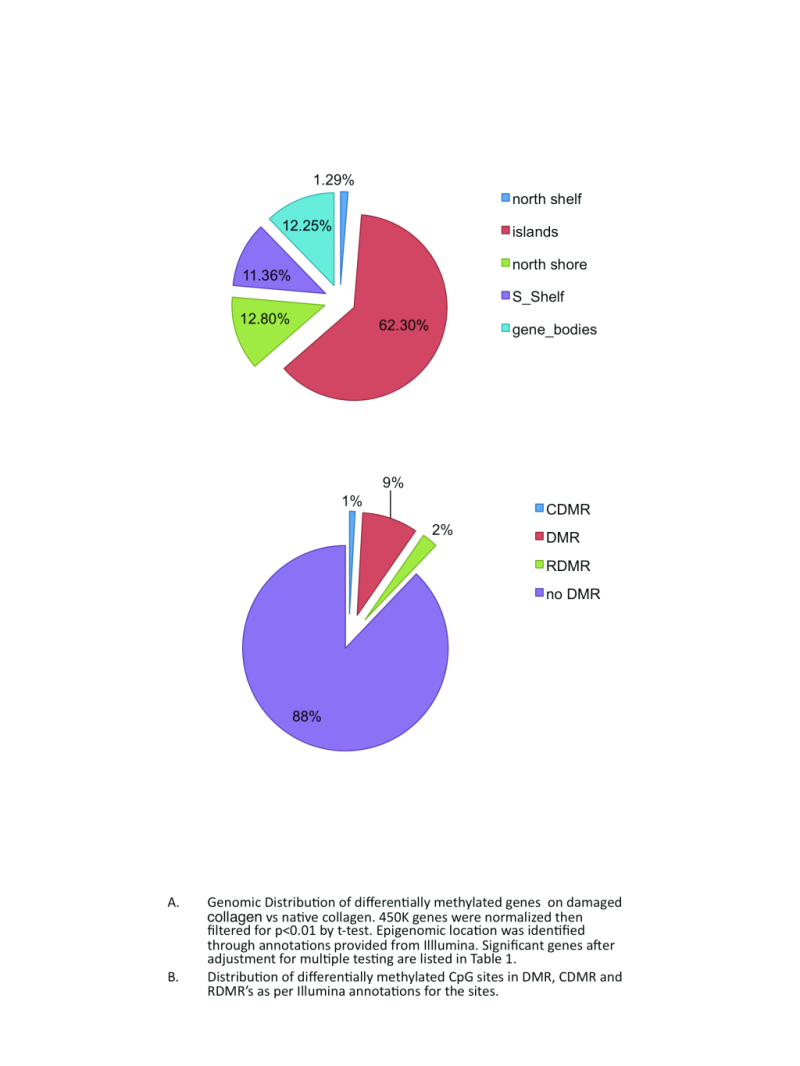

Supplement: Figure S3 — A. Genomic Distribution of differentially methylated genes on damaged collagen vs native collagen. 450K genes were normalized then filtered for p<0.01 by t-test. Epigenomic location was identified through annotations provided from Illlumina. Significant genes after adjustment for multiple testing (p<0.05) are listed in Table 1. B. Distribution of differentially methylated CpG sites in DMR, CDMR and RDMR's as per Illumina's annotations for the sites. (TIFF) [file pone.0069089.s003.tif]
